# Deletion of Adseverin in Osteoclasts Affects Cell Structure But Not Bone Metabolism

**DOI:** 10.1007/s00223-017-0271-6

**Published:** 2017-04-07

**Authors:** Yixuan Cao, Yongqiang Wang, Sara Sprangers, Daisy I. Picavet, Michael Glogauer, Christopher A. McCulloch, Vincent Everts

**Affiliations:** 1 Department of Oral Cell Biology and Functional Anatomy, Academic Centre for Dentistry Amsterdam (ACTA), Research Institute MOVE, University of Amsterdam and VU University Amsterdam, 11N-43, Gustav Mahlerlaan 3004, 1081 LA Amsterdam, The Netherlands; 2 Matrix Dynamics Group, Faculty of Dentistry, University of Toronto, Ontario, Canada; 3 Department of Cell Biology and Histology, Core Facility Cellular Imaging, Academic Medical Center, University of Amsterdam, Amsterdam, The Netherlands

**Keywords:** Adseverin, Osteoclast, Morphology, Transmission electron microscopy

## Abstract

Adseverin is an actin-severing/capping protein that may contribute to osteoclast differentiation in vitro but its role in bone remodeling of healthy animals is not defined. We analyzed bone and osteoclast structure in adseverin conditional null mice at alveolar and long bone sites. In wild-type and adseverin null mice, as measured by dual-energy X-ray absorptiometry, there were no differences of bone mineral content or bone mineral density, indicating no change of bone metabolism. In tibiae, TRAcP^+^ osteoclasts were formed in comparable numbers in adseverin null and wild-type mice. Ultrastructural analysis showed normal and similar abundance of ruffled borders, sealing zones, and mitochondria, and with no difference of osteoclast nuclear numbers. In contrast, analyses of long bone showed that in the absence of adseverin osteoclasts were smaller (120 ± 13 vs. 274 ± 19 µm^2^; *p* < 0.05), as were nuclear size and the surface area of cytoplasm. The nuclei of adseverin null osteoclasts exhibited more heterochromatin (31 ± 3%) than wild-type cells (8 ± 1%), suggesting that adseverin affects cell differentiation. The data indicate that in healthy, developing tissues, adseverin contributes to the regulation of osteoclast structure but not to bone metabolism in vivo.

## Introduction

Bone remodeling is a dynamic, life-long process in which bone formation by osteoblasts and bone resorption by osteoclasts are balanced to maintain a steady state. Osteoclasts are multinucleated resorptive cells of bone [1] that contribute to the maintenance of bone homeostasis. Osteoclasts are formed by fusion of monocyte/macrophage lineage precursor cells under the control of macrophage colony stimulating factor (M-CSF), which regulates the survival and proliferation of precursors [2], and by receptor activator of nuclear factor-kappa B ligand (RANKL), which promotes osteoclast differentiation and bone resorptive activity [3–5].

During osteoclastogenesis, the tight control of actin filament organization is crucial for cell formation, fusion, and differentiation and for cell attachment to bone surfaces. Notably, there are two structures in osteoclasts that are enriched with actin filaments: podosomes and sealing zones [6]. In early stages of osteoclastogenesis, podosomes are organized as clusters, which associate into actin rings that eventually form belts at the osteoclast periphery. Sealing zones form when osteoclasts adhere to mineralized bone surfaces, after which bone resorption is initiated [6, 7]. The organization and maintenance of these structures in osteoclasts is very dependent on the appropriate formation of actin filaments, which in turn requires temporally and spatially appropriate activities of actin-binding proteins.

There are hundreds of actin-binding proteins, which include the gelsolin family of actin-severing and actin-capping proteins that contribute to actin filament remodeling in a calcium-dependent manner [8, 9]. Gelsolin is involved in osteoclast function since compared to wild type, gelsolin-deficient mice exhibit increased bone mass and bone strength and defective podosome assembly in osteoclasts [10]. During osteoclastogenesis, gelsolin expression levels are relatively constant, while in cultured cells the expression of the gelsolin family protein adseverin is dramatically increased [11]. More recent data indicate that adseverin is important in osteoclastogenesis and may be an essential regulator of osteoclastic activity [11–13].

Adseverin (also known as scinderin) is an actin-severing/capping protein that has been studied in depth in chromaffin cells of the adrenal medulla [14]. The severing function of adseverin helps control actin filament length, while the actin-capping function of adseverin stabilizes actin filaments. Adseverin is highly expressed in many types of secretory cells, platelets [15], chondrocytes [16], odontoblasts [17], and differentiating osteoclasts [18]. Adseverin stimulates osteoclast differentiation and cell–cell fusion in cultured cells; the expression of adseverin is also increased in RANKL-induced osteoclastogenesis [11–13]. Adseverin knockdown inhibits bone resorption, reduces the secretion of TRAcP and cathepsin K, and alters actin filament organization [11, 13]. The increased expression of adseverin in osteoclastogenesis is dependent on the nuclear factor-kappa B (NF-κB) signaling pathway and on the expression of the osteoclastogenic transcription factor NFATc1 [13]. Adseverin co-localizes with actin filaments in podosomes; in adseverin knockout mice, osteoclasts exhibit lower number of podosome belts [12]. While these data suggest an important role for adseverin in osteoclastogenesis in vitro, it is not known whether adseverin impacts bone structure in healthy animals.

Here we examined bones from adseverin conditional null mice and background-matched wild-type mice by light microscopy, dual-energy X-ray absorptiometry (DEXA), and transmission electron microscopy (TEM). Since osteoclast function may differ in different skeletal sites [19–21], we compared osteoclast structure in alveolar bone with long bone and in healthy and inflamed sites.

## Methods and Materials

### Adseverin Knockout Mouse Model

All animal experiments were carried out in accordance with the Guide for the Humane Use and Care of Laboratory Animals and were approved by the University of Toronto Animal Care Committee. The generation of adseverin conditional knockout (adseverin KO) mice is described elsewhere [12]. In brief, mice harboring the conditional allele of adseverin were obtained by creation of a targeting vector that interrupted the expression of adseverin. After introduction of the targeting construct, LoxP floxed adseverin C57BL/6 mice were crossed with TRAcP-Cre C57 mice (from Christine M. Hachfeld, Mayo Clinic, Rochester, MN). The male mice generated by this breeding were further back-crossed with the pure LoxP floxed adseverin females. Littermates that did not express TRAcP-Cre are designated here as wild type (WT). The effectiveness of the knockout was confirmed by expression of TRAcP-Cre and LoxP by PCR. The primers that were used to genotype the adseverin mice were as follows: Cre expression: Forward: 5′-GAGTGATGAGGTTCGCAAGA-3′; Reverse: 5′-CTACACCAGAGACGGAAATC-3′; Product size: 635 bp. Distal LoxP: SCAD3: 5′-GTTAGTATTCCTCACTGGCACCC-3′; Ads-SDL2: 5′ ATGTTTCAGGACAGGAGTCTGAGC-3′; Presence of the distal LoxP: 363 bp; Wild-type adseverin allele: 289 bp. Around the left upper first molar, inflammation was induced by bacteria ligature in both WT and adseverin KO mice to compare the phenotype of that in the right part of the same mouse.

### Dual-Energy X-ray Absorptiometry

DEXA was performed on 3-month-old mice after CO_2_ asphyxiation, using an animal PIXImus densitometer (Lunar; GE). Data for bone mineral density (BMD) and bone mineral content (BMC) were collected for the lumbar vertebrae, right femur, and the entire skeleton after masking of the heads by a single operator.

### TRAcP Staining and Image Analysis

Distal tibia were removed, fixed with 4% paraformaldehyde (PFA), and decalcified in 10% EDTA (pH 7.4) at 4 °C for 2 weeks. After dehydration, the samples were embedded in paraffin and sectioned (5 µm thick). TRAcP staining was performed to assess the presence of osteoclasts. Osteoclasts were visualized with a Nikon Eclipse E1000 microscope and images were obtained with a Hamamatsu ORCA-ER camera and processed using Simple PCI software (Version 5.2.1.1609; Compix Inc). Osteoclasts were recognized as TRAcP^+^ cells and the number of osteoclasts was counted relative to the bone surface, according to standardized histomorphometric methods [22].

### Transmission Electron Microscopy

At 2 months of age, WT and adseverin KO mice were sacrificed. Mice were perfused with fixative (1% glutaraldehyde + 4% formaldehyde in 0.1 M sodium cacodylate buffer). Maxillae and long bones were collected and fixed in the same fixative. After decalcification in EDTA (pH = 7.2) for 14 days at room temperature, specimens were post-fixed with 1% OsO_4_ for 1 h. After subsequent washing in buffer, samples were dehydrated by an ethanol series. Specimens were embedded in epoxy resin (LX112). Resin-embedded specimens were trimmed and 1-µm thin sections were cut. Sections were stained with Richardson’s solution for light microscopic analysis (Leica DMRA). Image-Pro plus software (Media Cybernetics, Silver Spring, MD) was used to assess the size of osteoclasts.

After examining the sections by light microscopy, sites of interest were selected and ultrathin sections (80 nm) were cut with a diamond knife. The ultrathin sections were collected on Formvar-coated copper grids and counterstained with uranyl acetate and lead citrate. Micrographs were obtained with a Philips CM10 electron microscope. Size measurement of osteoclasts/nuclei/cytoplasm area was assessed by Image-Pro plus software (Media Cybernetics, Silver Spring, MD). Osteoclasts with ≥1 nucleus were used for analysis. Stereological analysis was performed according to Weibel et al. [23].

### Statistical Analysis

Results were shown as mean ± SD. Each experiment had a sample size of *n* ≥ 3, unless otherwise stated. Mean of the measurements of each osteoclast/nuclei/cytoplasm was calculated per mouse and pooled to compare the difference between WT and adseverin KO. Statistical analysis was performed using GraphPad Prism (version 5.0; GraphPad Software, LaJolla, CA). Student’s *t* test was used to compare the data between WT and KO. Statistical significance was set at *p* < 0.05.

## Results

### Osteoclast Number and Bone Resorption

**Fig. 1:**
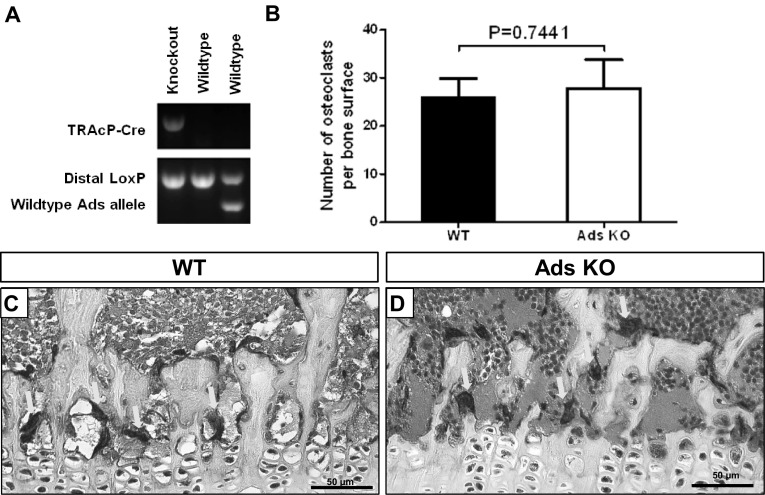
The number of osteoclasts in vivo was not changed by knocking out adseverin. **a** Mice were genotyped using PCR to test the effectiveness by determining TRAcP-Cre (635 bp), distal floxed Ads product (363 bp) as well as wild-type mouse Ads amplicon (289 bp). **b** No significant difference of osteoclast number per bone surface was found (*p* = 0.7441). **c, d** TRAcP-stained sections of distal tibiae were analyzed and TRAcP^+^ cells (*arrow*) were shown in WT (**c**) and adseverin KO (**d**) samples. *Scale bar* 50 µm

The effectiveness of conditional knockout of adseverin was confirmed by PCR (Fig. 1a). The role of adseverin in bone resorption in vivo is not well defined: previous micro-CT data showed no difference of bone structure between normal WT and adseverin KO mice [12], while in the same study adseverin null mice were protected from inflammation-mediated bone loss. To determine whether osteoclasts and their resorptive activity are dependent on adseverin, we analyzed TRAcP^+^ osteoclasts in long bones of WT and adseverin KO mice (Fig. 1c, d). The number of TRAcP^+^ osteoclasts per bone surface was not different between the two genotypes (Fig. 1b). DEXA analysis showed no difference of bone mineral density or bone mineral content of the whole body, vertebrae, or femurs between adseverin KO and WT mice at 3 months of age (Table 1). These data indicate that adseverin is not required for normal bone formation and remodeling.

**Table 1:** DEXA analysis of BMC and BMD of WT and adseverin KO mice

Samples	Parameters	WT	Adseverin KO	*p* value
3 months old (*n* = 6)	Whole body BMC (g)	0.391 ± 0.010	0.338 ± 0.020	0.060
	Whole body BMD (g/cm^2^)	0.060 ± 0.002	0.056 ± 0.001	0.069
	Vertebrae BMC (g)	0.053 ± 0.001	0.044 ± 0.006	0.163
	Vertebrae BMD (g/cm^2^)	0.060 ± 0.001	0.054 ± 0.004	0.261
	Femur BMC (g)	0.017 ± 0.001	0.016 ± 0.001	0.776
	Femur BMD (g/cm^2^)	0.066 ± 0.003	0.066 ± 0.003	0.877

No significant difference was found between WT and adseverin KO mice by DEXA analysis

Bone mineral content (BMC) and bone mineral density (BMD) were collected from the lumbar vertebrae, right femur, and the entire skeleton after masking of the heads

*p* values were calculated by *t* tests and data were shown as mean ± SD

*p* < 0.05 was considered statistically significant

### Osteoclast Morphology

#### Alveolar Bone

**Fig. 2:**
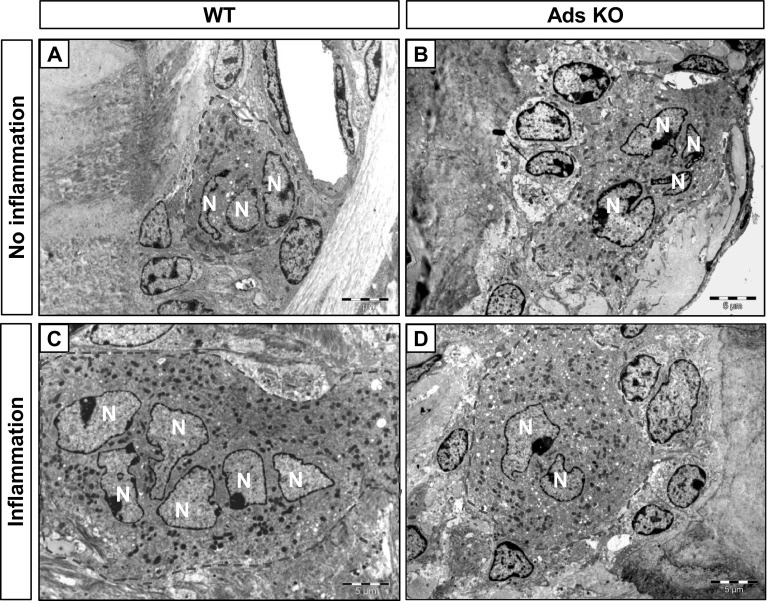
Morphology of osteoclasts in molar. Osteoclasts adjacent to the alveolar bone of molars were visualized by TEM and compared between WT and adseverin KO mice. **a** Normal condition (without ligature) in WT mice. **b** Normal condition in adseverin KO mice. **c** Inflammatory condition (induced with ligature around molar) in WT mice. **d** Inflammatory condition in adseverin KO mice. Osteoclasts are *encircled* with a *dashed line. N* nucleus. *Scale bar* 5 µm

Osteoclasts on the surface of the alveolar bone proper, and those located adjacent to the periodontal ligament, exhibited multiple nuclei and numerous mitochondria (Fig. 2). These features were similar for WT (Fig. 2a, c) and adseverin KO mice (Fig. 2b, d). In some samples in which silk ligatures were wrapped around the circumference of molar teeth to induce gingival inflammation, there were osteoclasts on the surface of the adjacent alveolar bone but there were no obvious differences of osteoclast structure between genotypes (Fig. 2c, d). Although there were abundant large osteoclasts on bone surfaces adjacent to inflamed gingival tissues, because of the low number of osteoclasts, we could not conduct meaningful statistical analysis of cell size.

#### Long Bone

**Fig. 3:**
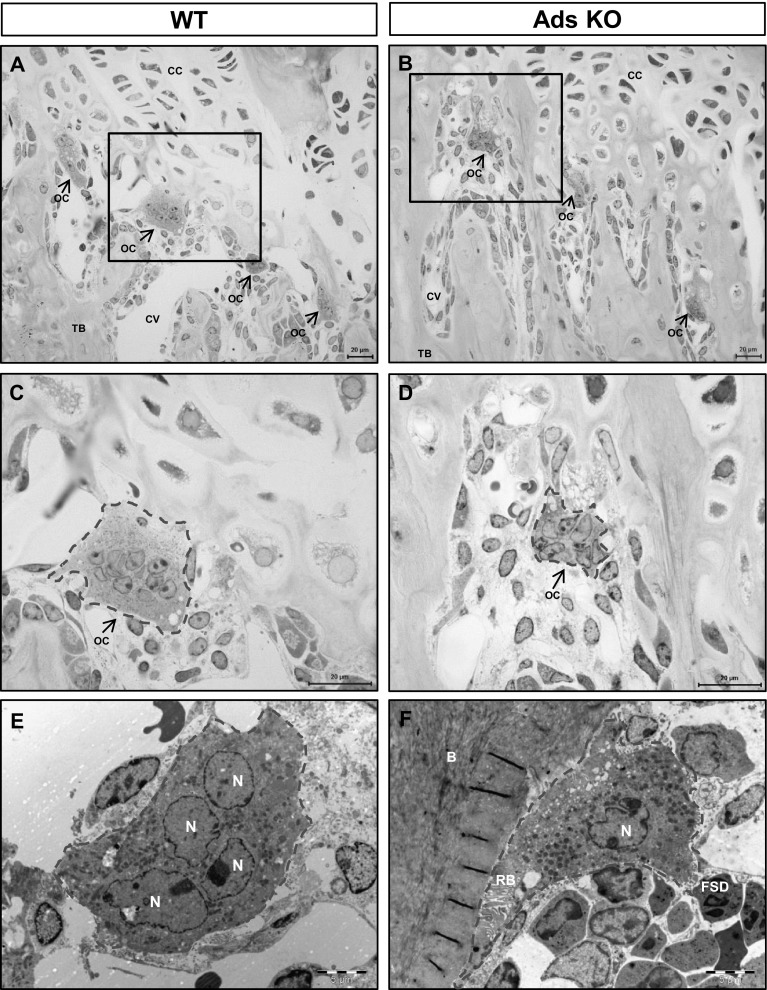
Morphology of osteoclasts in long bone. **a–d** Morphology of osteoclasts as observed by light microscopy. Relatively high numbers of osteoclasts were found in the growth plate area in both WT (**a, c**) and adseverin KO (**b, d**) mice. *Arrows* point to osteoclasts. *Scale bar* 20 µm. **e, f** Morphology of osteoclasts visualized by TEM. *Scale bar* 5 µm. Osteoclasts are *encircled* with a *dashed line. N* nucleus, *B* bone, *RB* ruffled border, *FSD* functional secretory domain, *OC* osteoclast, *CC* chondrocyte, *CV* capillary vessel, *TB* trabecular bone

We found large numbers of osteoclasts on bone and calcified cartilage surfaces adjacent to the growth plate of tibiae in WT and KO mice (Fig. 3a–d; see also Fig. 1). Most osteoclasts were found at the junction of the growth plate cartilage and trabecular bone and were always in close vicinity to blood vessel capillaries. There were no obvious ultrastructural differences between osteoclasts from WT mice and KO mice (Fig. 3e, f). Osteoclasts in WT and adseverin KO mice were tightly adherent to the bone surface and exhibited well-developed sealing zones and ruffled borders.

#### Ruffled Borders

**Fig. 4:**
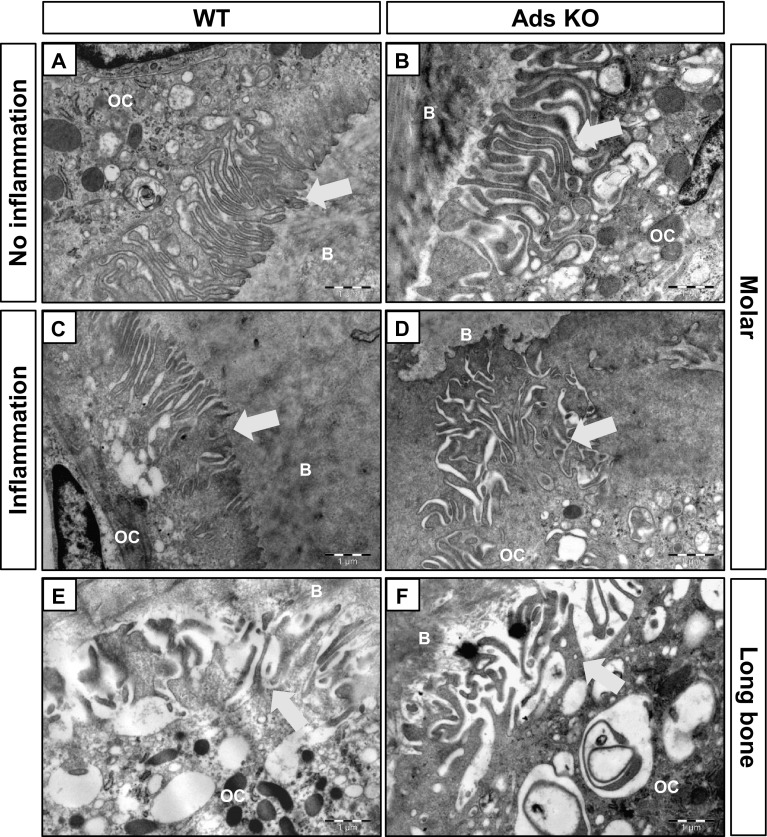
Osteoclasts of adseverin KO mice form a normal ruffled border. **a** Ruffled border of an osteoclast from molar in control condition in WT. **b** Ruffled border of an osteoclast from molar in control condition in adseverin KO mice. **c** Ruffled border of an osteoclast from molar in inflammatory condition in WT mice. **d** Ruffled border of an osteoclast from molar in inflammatory condition in adseverin KO mice. **e** Ruffled border of an osteoclast from long bone in WT. **f** Ruffled border of an osteoclast from long bone in adseverin KO mice. *Arrows* point to the ruffled border. *OC* osteoclast, *B* bone. *Scale bar* 1 µm

As adseverin may be important for podosome structure and bone resorption in vitro [12], we examined ruffled borders in detail. Ruffled borders were well developed in osteoclasts of both genotypes and were readily apparent in alveolar bone (Fig. 4a–d) and long bone (Fig. 4e, f) of WT and KO mice. There were no obvious morphological differences between the genotypes. Experimentally induced inflammation did not affect the formation of ruffled borders in WT (Fig. 4c) or adseverin KO mice (Fig. 4d). At healthy and inflamed sites, ruffled borders exhibited long, finger-like extensions (about 2 µm long, 100–200 nm wide) and were enriched with vacuoles that are important in exocytosis and endocytosis in bone resorption [24].

#### Sealing Zones

**Fig. 5:**
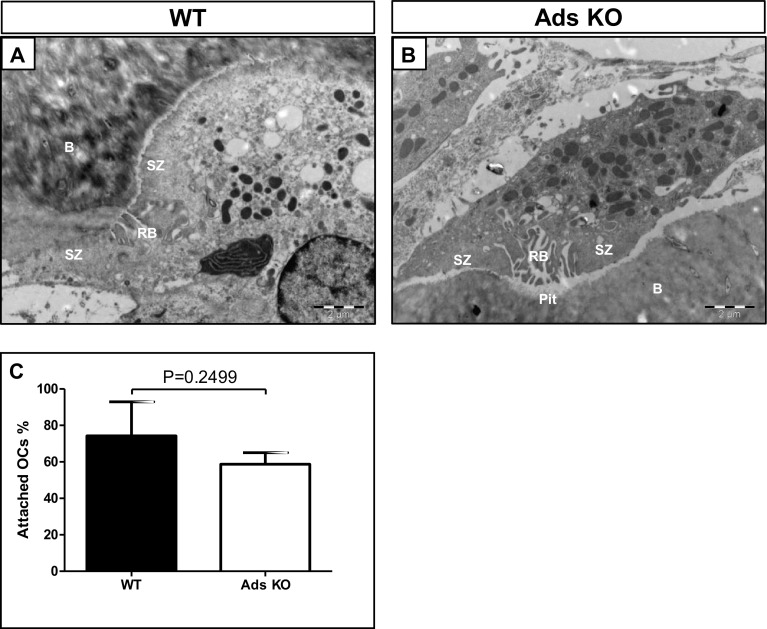
Adseverin knockout did neither affect the attachment nor the formation of sealing zone in vivo. **a, b**. Sealing zone structure in WT mice (**a**) and adseverin KO mice (**b**). **c** There was no significant difference of the attachment between WT and adseverin KO mice. *SZ* sealing zone, *RB* ruffled border, *B* bone. *Scale bar* 2 µm

Sealing zones were prominent in osteoclasts of WT and adseverin KO mice (Fig. 5a, b). The numbers of osteoclasts associated with bone surface were not different between the two genotypes (*p* = 0.25), indicating that adseverin does not contribute to osteoclast attachment to bone.

### Osteoclast Size

**Fig. 6:**
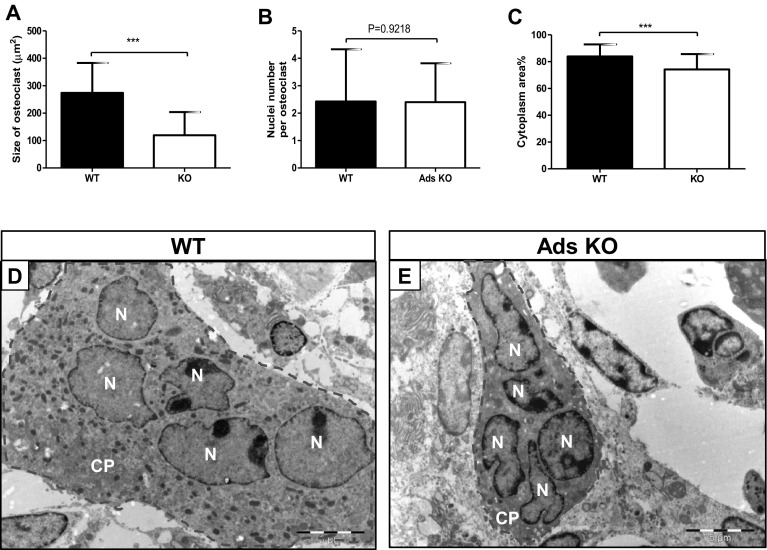
The size of osteoclasts and cytoplasm surface area were significantly decreased in adseverin KO mice in long bone. **a** Comparison of the size of osteoclasts between WT and adseverin KO mice (***p < 0.001). **b** The number of nuclei per osteoclast was similar in adseverin KO mice. **c** Percentage of cytoplasm surface area was significantly reduced in adseverin KO mice (***p < 0.001). Assessment of size was performed by Image-Pro Plus and the number of osteoclasts analyzed for the size was 63 for WT, and 134 for adseverin KO; for # cytoplasm surface area 32 for WT and 42 for adseverin KO. **d, e** An example of an osteoclast in WT (**d**) and adseverin KO (**e**) mice. Osteoclasts are *encircled* by a *dashed line. N* nucleus, *B* bone. *Scale bar* 5 µm

Previous in vitro studies showed that osteoclasts generated from precursors derived from adseverin null mice were smaller [12]. In the present study, we analyzed the size of osteoclasts formed in vivo and found that adseverin KO mice had significantly smaller osteoclasts (Fig. 6). Osteoclasts in WT mice were 274 ± 19 µm^2^, while osteoclasts in adseverin KO mice were less than half of this size (120 ± 13 µm^2^; *p* < 0.001; Fig. 6a). Since osteoclast size is affected by cell fusion processes, the number of nuclei per cell was assessed. There was no difference in the numbers of nuclei per cell (Fig. 6b), indicating that adseverin does not affect the fusion of osteoclast precursor cells in normal bone in vivo. Further, consistent with their smaller size, osteoclasts from adseverin null mice exhibited smaller cytoplasmic area (Fig. 6c).

### Nuclear Size and Chromatin Distribution

**Fig. 7:**
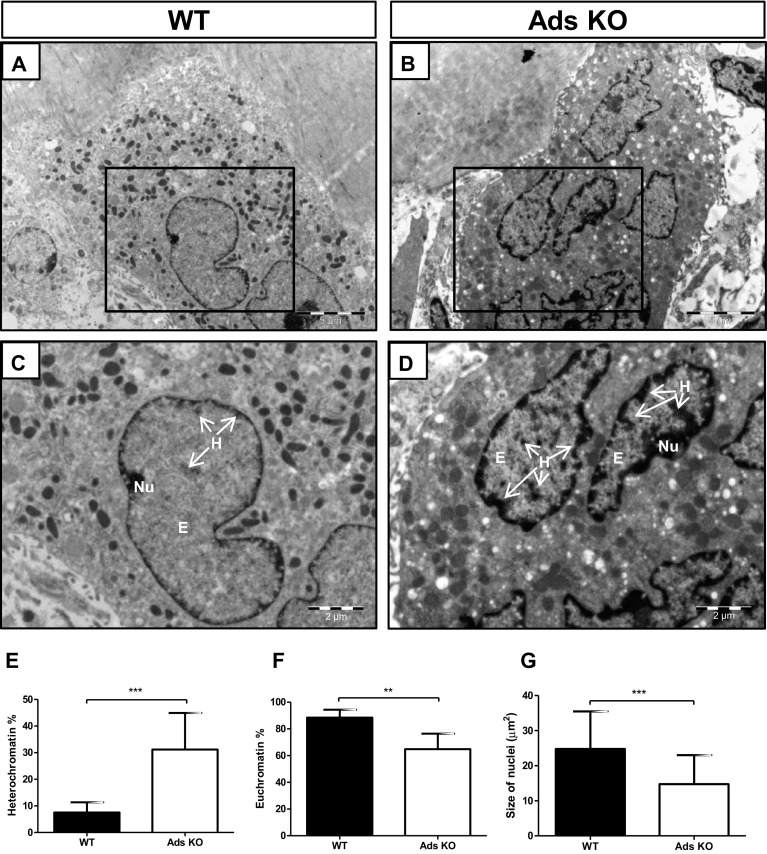
Nuclei of osteoclasts in adseverin knockout mice contained more heterochromatin and were smaller. **a–d** Nuclei of osteoclasts in WT (**a, c**) and adseverin KO mice (**b, d**). **a, b** *Scale bar* 5 µm; **c, d** *scale bar* 2 µm. **e** Percentage of heterochromatin was significantly higher in nuclei from adseverin KO mice. **f** Percentage of euchromatin was significantly lower in nuclei from adseverin KO mice. **g** The size of nuclei proved to be smaller in adseverin KO mice. Assessment of size was performed by Image-Pro Plus and the number of nuclei analyzed was 52 for WT and 67 for adseverin KO. *E* euchromatin, *H* heterochromatin, *Nu* nucleolus (**p < 0.01, ***p < 0.001)

Overall assessment of the shapes of osteoclast nuclei in adseverin KO mice indicated marked differences between KO and wild-type mice (Fig. 7a–d). Osteoclast nuclei in WT mice were predominantly round or oval shaped with a smooth, nuclear membrane surface (Fig. 7a, c), while the nuclei of osteoclasts in adseverin KO mice exhibited prominent folds and indentations (Fig. 7b, d).

The spatial distributions of euchromatin and heterochromatin were affected by adseverin expression. The amount of heterochromatin, which is a tightly packed, electron-dense, transcriptionally inactive chromatin, was fourfold more abundant in osteoclast nuclei of adseverin KO mice (31 ± 3%) than WT mice (8 ± 1%) (Fig. 7e). Euchromatin, which is an electron-lucent, loosely packed transcriptionally active chromatin, showed the opposite distribution (Fig. 7f). Examination of nuclear size showed that osteoclast nuclei in adseverin KO mice were smaller than osteoclast nuclei in WT mice (Fig. 7g).

## Discussion

Our findings indicate that the actin-capping/severing protein adseverin contributes to the maintenance of osteoclast size and nuclear chromatin organization. In the absence of adseverin expression, osteoclasts were smaller, and with reduced cytoplasmic area and nuclear size, suggesting that osteoclast structure is affected by adseverin-dependent regulation of the actin filament network. Indeed, previous studies indicated that actin filaments could control the volume [25] and growth of cells [26]. The actin cytoskeleton is a dynamic system that may serve as an interactive sensor in cell volume regulation to maintain actin filaments in equilibrium [27]. Actin filament organization may be linked to the extent of cell swelling, by either regulation of ion transport systems or interactions of actin-associated proteins with discrete plasma membrane domains [28]. Cantiello and colleagues suggested that interactions between actin and actin-severing/capping proteins influence the activity of Na^+^/K^+^ channels, which regulate the movement of water and affect cell volume [29]. We propose that when adseverin is not expressed, the organization of actin cytoskeletal structures is affected, which in turn affects osteoclast size but, remarkably, not podosome structure. Evidently, the stability and turnover of actin filament structures in osteoclasts is under the control of multiple regulatory systems, one of which is adseverin.

The reduced size of osteoclasts in vivo in adseverin KO mice is consistent with previous in vitro studies showing that osteoclasts generated from precursor cells obtained from adseverin null mice were smaller [11, 12]. The smaller size of these osteoclasts was considered to be a result of the impaired fusion capacity of osteoclast precursors. Our current data show that the number of nuclei per osteoclast did not differ between adseverin null and wild-type littermates, indicating that fusion was not affected in vivo.

Although actin is one of the most abundant proteins in the cytoplasm, new data show a role for nuclear actin in cell function and structure [30–32]. While adseverin has not been demonstrated in the nucleus, gelsolin [31] and the actin-capping protein CapG [33] have been reported in the nucleus. Actin is present in cells in globular (G-actin), oligomeric, or filamentous form. In the nucleus, actin is mainly found as G-actin or oligomers [34], which requires the action of severing proteins like gelsolin to generate short oligomers or monomers. Nuclear actin assembly can affect transcription [35], suggesting an important relationship between the organization of actin filaments and transcriptional regulation. Consistent with this notion, we found that in the absence of adseverin the packing of heterochromatin was increased. Since heterochromatin is thought to be transcriptionally inactive because of a high proportion of repetitious sequences [36, 37], adseverin may regulate transcription and potentially histone assembly, which is reflected in chromatin packing. Adseverin, possibly in combination with actin, may play a previously unrecognized role in the organization of chromatin in osteoclast nuclei.

One of the most prominent sites of actin cytoskeletal organization in the osteoclast is at the sealing zone, where cells are firmly attached to the bone surface, which is a prerequisite for efficient bone degradation [7]. While previous in vitro studies showed that adseverin affects podosome self-organization [12], we found no differences in the structure of sealing zones of osteoclasts between WT and adseverin KO mice. Further, osteoclasts in adseverin KO mice formed a normal ruffled border and with no effect on bone resorption compared with WT mice. These data are in agreement with a previous study in which micro-CT showed an unchanged bone phenotype in healthy adseverin KO mice [12]. We analyzed osteoclasts at different skeletal sites and compared normal bone with bone adjacent to sites with induced inflammation. The structure and function of osteoclasts from different skeletal sites can be quite different [19]. In vitro studies showed that osteoclasts generated from precursors derived from alveolar bone and from long bone exhibit marked differences in cell shape [20]. Here we found no measureable differences in osteoclasts at long bone and alveolar bone sites in WT and adseverin null conditional mice. Further studies are needed to elucidate this difference between in vitro and in vivo works.

Collectively, our findings indicate that although the actin-severing/capping protein, adseverin, does not contribute to bone metabolism in vivo, it plays an important role in the control of osteoclast size and in the organization of chromatin structure and nuclear size.

## References

[1] Hayashi S, Yamane T, Miyamoto A (1998). Commitment and differentiation of stem cells to the osteoclast lineage. Biochem Cell Biol.

[2] Fuller K, Owens JM, Jagger CJ (1993). Macrophage colony-stimulating factor stimulates survival and chemotactic behavior in isolated osteoclasts. J Exp Med.

[3] Lacey DL, Timms E, Tan HL (1998). Osteoprotegerin ligand is a cytokine that regulates osteoclast differentiation and activation. Cell.

[4] Hsu H, Lacey DL, Dunstan CR (1999). Tumor necrosis factor receptor family member RANK mediates osteoclast differentiation and activation induced by osteoprotegerin ligand. Proc Natl Acad Sci USA.

[5] Asagiri M, Takayanagi H (2007). The molecular understanding of osteoclast differentiation. Bone.

[6] Jurdic P, Saltel F, Chabadel A, Destaing O (2006). Podosome and sealing zone: specificity of the osteoclast model. Eur J Cell Biol.

[7] Saltel F, Chabadel A, Bonnelye E, Jurdic P (2008). Actin cytoskeletal organisation in osteoclasts: a model to decipher transmigration and matrix degradation. Eur J Cell Biol.

[8] Silacci P, Mazzolai L, Gauci C (2004). Gelsolin superfamily proteins: key regulators of cellular functions. Cell Mol Life Sci.

[9] Sakurai T, Kurokawa H, Nonomura Y (1991). Comparison between the gelsolin and adseverin domain structure. J Biol Chem.

[10] Chellaiah M, Kizer N, Silva M (2000). Gelsolin deficiency blocks podosome assembly and produces increased bone mass and strength. J Cell Biol.

[11] Hassanpour S, Jiang H, Wang Y (2014). The actin binding protein adseverin regulates osteoclastogenesis. PLoS ONE.

[12] Jiang H, Wang Y, Viniegra A (2015). Adseverin plays a role in osteoclast differentiation and periodontal disease-mediated bone loss. FASEB J.

[13] Song M-K, Lee ZH, Kim H-H (2015). Adseverin mediates RANKL-induced osteoclastogenesis by regulating NFATc1. Exp Mol Med.

[14] Hartwig JH, Kwiatkowski DJ (1991). Actin-binding proteins. Curr Opin Cell Biol.

[15] Zunino R, Li Q, Rosé SD (2001). Expression of scinderin in megakaryoblastic leukemia cells induces differentiation, maturation, and apoptosis with release of plateletlike particles and inhibits proliferation and tumorigenesis. Blood.

[16] Nurminsky D, Magee C, Faverman L, Nurminskaya M (2007). Regulation of chondrocyte differentiation by actin-severing protein adseverin. Dev Biol.

[17] Li X, Jiang H, Huang Y (2015). Expression and function of the actin-severing protein adseverin in the proliferation, migration, and differentiation of dental pulp cells. J Endod.

[18] Yang G, Zaidi M, Zhang W (2008). Functional grouping of osteoclast genes revealed through microarray analysis. Biochem Biophys Res Commun.

[19] Everts V, de Vries TJ, Helfrich MH (2009). Osteoclast heterogeneity: lessons from osteopetrosis and inflammatory conditions. Biochim Biophys Acta.

[20] Azari A, Schoenmaker T, de Souza Faloni AP (2011). Jaw and long bone marrow derived osteoclasts differ in shape and their response to bone and dentin. Biochem Biophys Res Commun.

[21] Jansen IDC, Mardones P, Lecanda F (2009). Ae2(a,b)-deficient mice exhibit osteopetrosis of long bones but not of calvaria. FASEB J.

[22] Dempster DW, Compston JE, Drezner MK (2013). Standardized nomenclature, symbols and units for bone histomorphometry: a 2012 update of the report of the ASBMR Histomorphometry Nomenclature Committee. J Bone Miner Res.

[23] Weibel ER, Kistler GS, Scherle WF (1966). Practical stereological methods for morphometric cytology. J Cell Biol.

[24] Lucht U (1972). Cytoplasmic vacuoles and bodies of the osteoclast: an electron microscope study. Z Zellforsch Mikrosk Ana.

[25] Henson JH (1999). Relationships between the actin cytoskeleton and cell volume regulation. Microsc Res Tech.

[26] Bunnell TM, Burbach BJ, Shimizu Y, Ervasti JM (2011). β-Actin specifically controls cell growth, migration, and the G-actin pool. Mol Biol Cell.

[27] Papakonstanti EA, Vardaki EA, Stournaras C (2000). Actin cytoskeleton: a signaling sensor in cell volume regulation. Cell Physiol Biochem.

[28] Mills JW, Schwiebert EM, Stanton BA (1994). Evidence for the role of actin filaments in regulating cell swelling. J Exp Zool.

[29] Cantiello HF (1995). Actin filaments stimulate the Na(+)–K(+)-ATPase. Am J Physiol.

[30] Chen M, Shen X (2007). Nuclear actin and actin-related proteins in chromatin dynamics. Curr Opin Cell Biol.

[31] Ocampo J, Mondragón R, Roa-Espitia AL (2005). Actin, myosin, cytokeratins and spectrin are components of the guinea pig sperm nuclear matrix. Tissue Cell.

[32] Castano E, Philimonenko VV, Kahle M (2010). Actin complexes in the cell nucleus: new stones in an old field. Histochem Cell Biol.

[33] Bettinger BT, Gilbert DM, Amberg DC (2004). Actin up in the nucleus. Nat Rev Mol Cell Biol.

[34] Kapoor P, Shen X (2014). Mechanisms of nuclear actin in chromatin remodeling complexes. Trends Cell Biol.

[35] Serebryannyy LA, Parilla M, Annibale P (2016). Persistent nuclear actin filaments inhibit transcription by RNA polymerase II. J Cell Sci.

[36] Richards EJ, Elgin SCR (2002). Epigenetic codes for heterochromatin formation and silencing: rounding up the usual suspects. Cell.

[37] Huisinga KL, Brower-Toland B, Elgin SCR (2006). The contradictory definitions of heterochromatin: transcription and silencing. Chromosoma.

